# Unraveling virulence determinants in extended-spectrum beta-lactamase-producing *Escherichia coli* from East Africa using whole-genome sequencing

**DOI:** 10.1186/s12879-023-08579-0

**Published:** 2023-09-07

**Authors:** Ivan Sserwadda, Benson R. Kidenya, Stephen Kanyerezi, Inyasi Lawrence Akaro, Baraka Mkinze, Stephen E. Mshana, Suhaila O. Hashim, Everlyne Isoe, Jeremiah Seni, Moses L. Joloba, Gerald Mboowa

**Affiliations:** 1 Department of Immunology and Molecular Biology, School of Biomedical Sciences, College of Health Sciences, Makerere University, P.O Box 7072, Kampala, Uganda; 2 Department of Biochemistry and Bioinformatics, School of Pure and Applied Sciences, Pwani University, P.O Box 195-80108, Kilifi, Kenya; 3 The African Center of Excellence in Bioinformatics and Data-Intensive Sciences, Infectious Diseases Institute, College of Health Sciences, Makerere University, P.O Box 22418, Kampala, Uganda; 4 Department of Biochemistry and Molecular Biology, Weill Bugando School of Medicine, Catholic University of Health and Allied Sciences, Mwanza, Tanzania; 5 Department of Surgery, Weill Bugando School of Medicine, Catholic University of Health and Allied Sciences, Mwanza, Tanzania; 6 Department of Microbiology and Immunology, Weill Bugando School of Medicine, Catholic University of Health and Allied Sciences, Mwanza, Tanzania; 7 Africa Centres for Disease Control and Prevention, African Union Commission, Roosevelt Street, P.O. Box 3243, Addis Ababa, W21 K19 Ethiopia

**Keywords:** Extended-spectrum β-lactamase, East Africa, Whole-genome sequencing, Antimicrobial resistance, Virulence factors

## Abstract

*Escherichia coli* significantly causes nosocomial infections and rampant spread of antimicrobial resistance (AMR). There is limited data on genomic characterization of extended-spectrum *β*-lactamase (ESBL)-producing *E. coli* from African clinical settings. This hospital-based longitudinal study unraveled the genetic resistance elements in ESBL *E. coli* isolates from Uganda and Tanzania using whole-genome sequencing (WGS). A total of 142 ESBL multi-drug resistant *E. coli* bacterial isolates from both Tanzania and Uganda were sequenced and out of these, 36/57 (63.1%) and 67/85 (78.8%) originated from Uganda and Tanzania respectively. Mutations in *RarD*, *yaaA* and *ybgl* conferring resistances to chloramphenicol, peroxidase and quinolones were observed from Ugandan and Tanzanian isolates. We reported very high frequencies for *bla*_CTX−M−15_ with 11/18(61.1%), and *bla*_CTX−M−27_ with 12/23 (52.1%), *bla*_TEM−1B_ with 13/23 (56.5%) of isolates originating from Uganda and Tanzania respectively all conferring resistance to Beta-lactam-penicillin inhibitors. We observed chloramphenicol resistance-conferring gene *mdfA* in 21/23 (91.3%) of Tanzanian isolates. Extraintestinal *E. coli* sequence type (ST) 131 accounted for 5/59 (8.4%) of Tanzanian isolates while enterotoxigenic *E. coli* ST656 was reported in 9/34 (26.4%) of Ugandan isolates. Virulence factors originating from *Shigella dysenteriae* Sd197 (*gspC, gspD, gspE, gspF, gspG, gspF, gspH, gspI*), *Yersinia pestis* CO92 (*irp1, ybtU, ybtX, iucA*), *Salmonella enterica* subsp. enterica serovar Typhimurium str. LT2 (*csgF* and *csgG*), and *Pseudomonas aeruginosa* PAO1 (*flhA, fliG*, *fliM*) were identified in these isolates. Overall, this study highlights a concerning prevalence and diversity of AMR-conferring elements shaping the genomic structure of multi-drug resistant *E. coli* in clinical settings in East Africa. It underscores the urgent need to strengthen infection-prevention controls and advocate for the routine use of WGS in national AMR surveillance and monitoring programs.

Availability of WGS analysis pipeline: the rMAP source codes, installation, and implementation manual can free be accessed via https://github.com/GunzIvan28/rMAP.

## Background

The antimicrobial resistance (AMR) phenomenon has spread rapidly over the course of the past decades to establish itself as a major global public health threat [1] in spite of the strides made by modern medicine to apply the use of antibiotics to ensure safe surgical procedures and improve the quality of medical care that have greatly reduced morbidity and mortality in public health [2, 3]. The bacterial infections that have been able to exhibit AMR have become fatal and given birth to a possible post-antibiotic era which initially was thought to be an apocalyptic fantasy before the 21st century [2, 4]. It goes without saying that the role played by humans in exacerbating the rate at which these infectious agents have developed resistance to antibiotics has been at the forefront of accelerating this phenomenon mainly through the rampant and inappropriate use of antibiotics [4, 5]. A previous study predicted an alarming 10 million deaths per annum with $100 trillion dollars’ worth of efforts trying to combat AMR by 2050 if this is not tackled [6]. Some regions in the world especially the African continent characterized by high infectious disease burdens and limited healthcare infrastructure have the least accurate and reliable statistical data on the epidemiology and impact of AMR on the public health sectors [7–9]. The rapid recent evolution of genomics-based technologies applied in the diagnosis and surveillance of the epidemiology of drug-resistant bacteria has led to the generation of large amounts of genomic data that have given deeper insights into the nature and changes of AMR determinants using modern bioinformatics analysis pipelines [10]. Application of next-generation sequencing technologies (NGS) alongside conventional microbiology procedures and antimicrobial susceptibility testing (AST) may be the key to understanding AMR [11], accelerating knowledge generation, and deploying interventions tailored towards optimization of antimicrobial use in public health [12].

Therefore, this study explored and sought to understand the epidemiology, and factors driving AMR in hospital settings in Uganda and Tanzania through the use of whole-genome sequencing (WGS) data combined with the socio-demographic metadata provided by the mother study.

## Materials and methods

### Study design and settings

This study utilized a laboratory-based and longitudinal study design approach. The laboratory-based design was used to undertake WGS to determine the AMR elements from the bacterial isolates.

### Study sites

This study was carried out at the orthopedic units of Mulago National Referral Hospital, Kampala, Uganda with geographical coordinates (0°20’16.0"N, 32°34’32.0"E) and Bugando Medical Centre (BMC), Mwanza, Tanzania with geographical coordinates (2°31’41.0"S, 32°54’27.0"E).

### Study population

The study population constituted of whole-genome sequence data obtained from a total of 142 multi-drug resistant *E. coli* bacterial isolates provided by the mother study titled, “Understanding Transmission Dynamics and Acquisition of Antimicrobial Resistance at Referral Hospitals and Community Settings in East Africa using Conventional Microbiology and Whole-Genome Sequencing”. The multi-drug isolates were collected from both study sites in Uganda (n = 57) and Tanzania (n = 85) from patients, the immediate non-medical caretakers of these patients, the immediate health workers attending to these patients and the patients’ environment as previously discussed in detail by the recent publications from Uganda [1] and Tanzania [13].

### Data collection and analysis tools

The WGS data used in this study was provided by a bigger mother study titled, “Understanding Transmission Dynamics and Acquisition of Antimicrobial Resistance at Referral Hospitals and Community settings in East Africa using Conventional Microbiology and Whole-Genome Sequencing (Grant number GCA/AMR/rnd2/058)”. The bacterial isolates were shipped and sequenced by the Earlham Institute, Norwich, located in the United Kingdom following the Low Input, Transposase Enabled (LITE) Illumina protocol using the Illumina NovaSeq 6000 System.

The analysis of WGS data was done using our previously published Linux command line-based bioinformatics workflow called “rMAP”, the Rapid Microbial Analysis pipeline [10]. Briefly, the whole-genome raw sequences together with the GenBank *Escherichia coli* str. K-12 substr. MG1655 with Accession NC_000913 reference was fed into the rMAP pipeline. Sequences in the format fastq.gz were used as the input for the pipeline. All sequences were inspected for quality in the rMAP pipeline [10] before any subsequent processes using the embedded FastQC [14] to generate individual sample reports and MultiQC [15] for aggregating all the multiple reports into one report. Adapters were trimmed off the sequences using Trimmomatic [16] with the selected parameters including minimum length and phred score set to 200 and 32 respectively.

The trimmed reads were loaded into the Shovill [17] *de-novo* pipeline using the Skesa as the assembler of choice. *K*-mer sizes of 31, 55, 79, 103 and 127 were used to determine the optimum genome assembly. Pilon was used for checking assembly errors, correcting ambiguous gaps, insertions, deletions and finally polishing the genomes [18]. Genome annotations were performed using the Prokka tool [19].

Single nucleotide polymorphism (SNP) variant calling was performed using SAMtools, Burrows-Wheeler Aligner (BWA), SAMclip, Freebayes and SnpEff [20–22]. The trimmed reads were aligned against an indexed reference in fasta format (GenBank reference *Escherichia coli* strain K-12 sub strain MG1655 Accession: NC_000913.3) using Burrows-Wheeler aligner [21] to produce Sequence Alignment Map (SAM) files. Soft and hard clipped alignments were removed from the SAM files using SAMclip(https://github.com/tseemann/samclip). SAMtools [20] then sorted, marked duplicates and indexed the resultant Binary Alignment Map (BAM) files. Freebayes [23]was used to call variants using Bayesian models to produce variant call format (VCF) files containing SNP information which was filtered using BCFtools(https://github.com/samtools/bcftools) and normalized of biallelic regions using Vt [24]. The filtered VCF files were annotated using snpEff [22]. Missense variants that were associated with resistance were identified from the VCF files according to their respective sites. Only true SNPs were considered for the downstream analysis; insertions, deletions, and complex SNPs were filtered out from the resistance-associated SNPs.

Phylogenetic inference by maximum likelihood was performed using MAFFT, IQtree, Vcf2phylip, and BMGE [25–28]. The rMAP pipeline collated all the individual VCF files into a single VCF containing all the samples and their SNPs before being transposed by vcf2phylip [26] into a multi-alignment fasta file. MAFFT software package was used to perform multiple sequence alignment [27]; removal of ambiguously aligned reads as well as extraction of informative sites was performed to infer phylogeny using BMGE [25]. IQtree [28]was then used to test various substitution models and construct trees from the alignments using the maximum-likelihood method in 1,000 bootstraps. The resulting trees were visualized in the form of rectangular phylograms.

Mass screening for AMR genes against CARD [29], ARG-ANNOT [30], NCBI, ResFinder, and MEGARES [31] databases was performed for each of the study isolates using the Abricate tool (https://github.com/tseemann/abricate). For consistency purposes, we compared results from the two most commonly used well-annotated AMR databases across the *E. coli* isolates (CARD and ResFinder) from both study sites. From our findings, we found more AMR genes conforming to > 90% cut-off for both coverage and identity being detected from the ResFinder database which were then presented in form frequencies and heatmaps in the results section.

Multi-locus sequence typing was performed using MLST (https://github.com/tseemann/mlst) from the *E. coli* assembled contigs. The *E. coli* isolates were determined against a set of seven (7) *E. coli* housekeeping genes (*adk*4, *fum*26, *gyrB*2, *icd*25, *mdh*5, *purA*5, and *recA*19).

Virulence factors were determined against the virulence factor database (VFDB) [32] using the Abricate tool (https://github.com/tseemann/abricate) to identify elements that conformed to > 90% cut-off for both coverage and identity.

## Results

### Pre-sequencing quality control

**Fig. 1:**
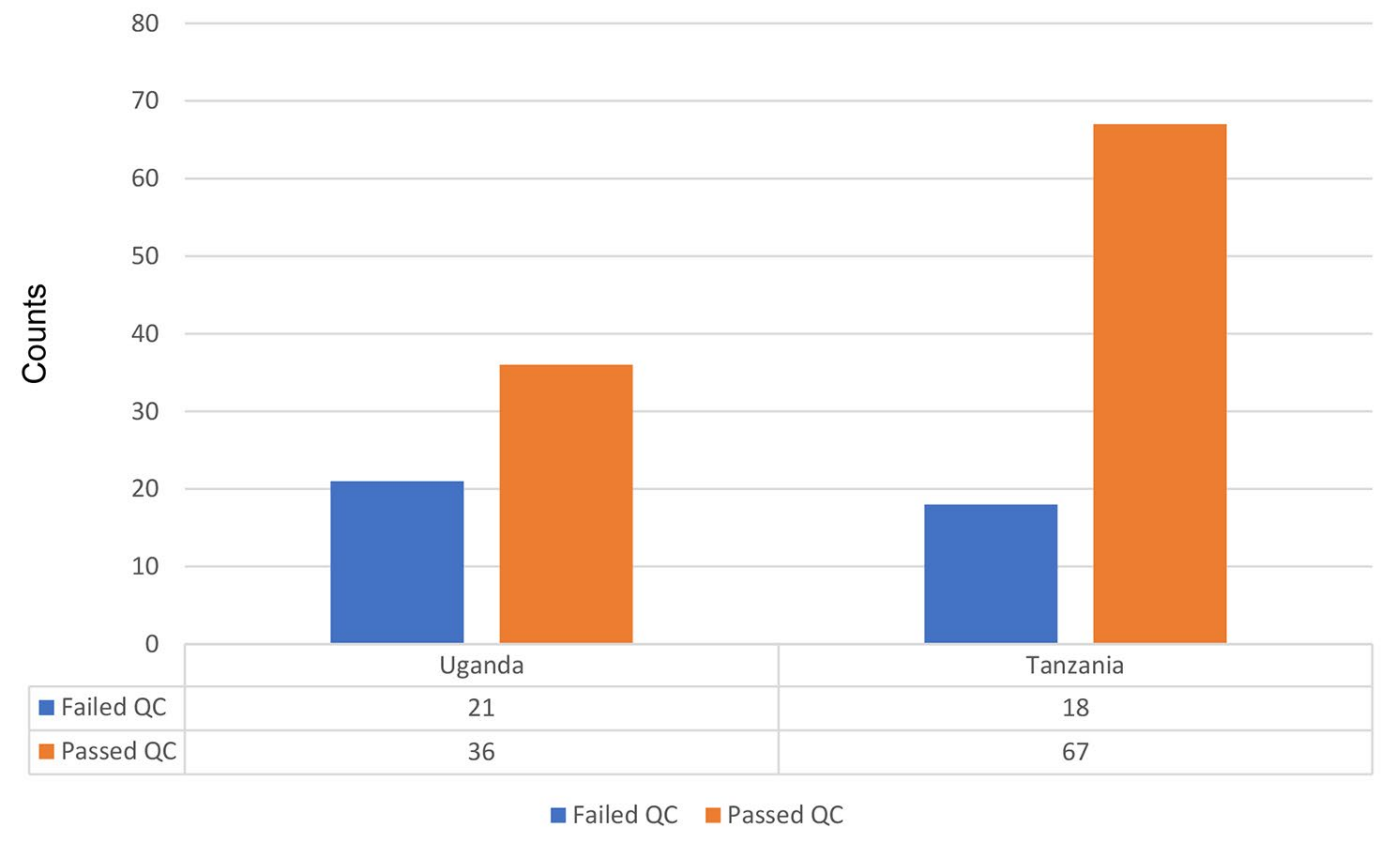
Proportions of the ESBL-producing *E. coli* from the study sites that conformed to initial pre-sequencing (LITE) Illumina protocol

A total of 142 ESBL multi-drug resistant *E. coli* bacterial isolates from both Tanzania and Uganda were sequenced. Only 36/57 (63.1%) of the ESBL-producing *E. coli* from Uganda and 67/85 (78.8%) of the ESBL-producing *E. coli* isolates from Tanzania passed the initial pre-sequencing Transposase Enabled (LITE) Illumina protocol and were selected for the downstream bioinformatics analysis as shown in Fig. 1.

### Raw sequence quality assessment, adapter, and poor read sequence trimming

The average read length of the sequences was 150 bp with an average Q-score of 32 as discussed in the methods section.

### De-novo genome assembly

Considering the main genome assembly parameters, the samples from the two study sites had an average of 132 bp (base pairs) for the mean read length and 525 bp for the contigs. An estimated average genome length of 5,138,608 bp, average GC-content of 51%, and average sequencing depth of 9.34X were reported for the isolates. A summary of the average genome assembly statistics across all analyzed samples is shown in Table 1.

**Table 1:** *De novo* genome assembly metrics and statistics for the study isolates

Metric	Uganda	Tanzania
Trimmed read counts	359,277	405,411
Mean Read Length (base pairs, bp)	133	131
Number of Contigs (> 200 bp)	612	437
Approximates Genome Length (bp)	5,034,750	5,242,466
Largest Contig (bp)	240,554	340,237
N50	80,279	116,894
GC- Content (%)	51.051	51.046
Depth (X)	8.85	9.822

### Single nucleotide polymorphism (SNP) variant calling

The annotated VCF files were interrogated for SNPs that were “missense” meaning those that altered the function of the protein and had a keyword “resistance”. Generally, the most clinically significant predominant resistance-associated SNPs and respective genes affected included: peroxide stress resistance protein (*yaaA*), chloramphenicol resistance permease (*RarD*), and quinolone resistance protein (*ybgl*). A detailed depiction of the distribution of these SNPs between the two study sites is shown in Table 2.

**Table 2:** Frequency of resistance-associated SNPs identified from Ugandan and Tanzanian isolates

Resistance gene	Functional annotation and conferred resistance	Uganda (*n* = 36)	Tanzania (*n* = 67)
*RarD*	Chloramphenicol resistance permease	14/36 (38.8%)	23/67 (34.3%)
*yaaA*	Peroxide stress resistance protein	11/36 (30.5%)	33/67 (49.2%)
*ybgl*	Quinolone resistance protein and radiation resistance protein	25/36 (69.4%)	30/67 (44.7%)

### Antimicrobial resistance gene determinants from Uganda and Tanzania

**Fig. 2:**
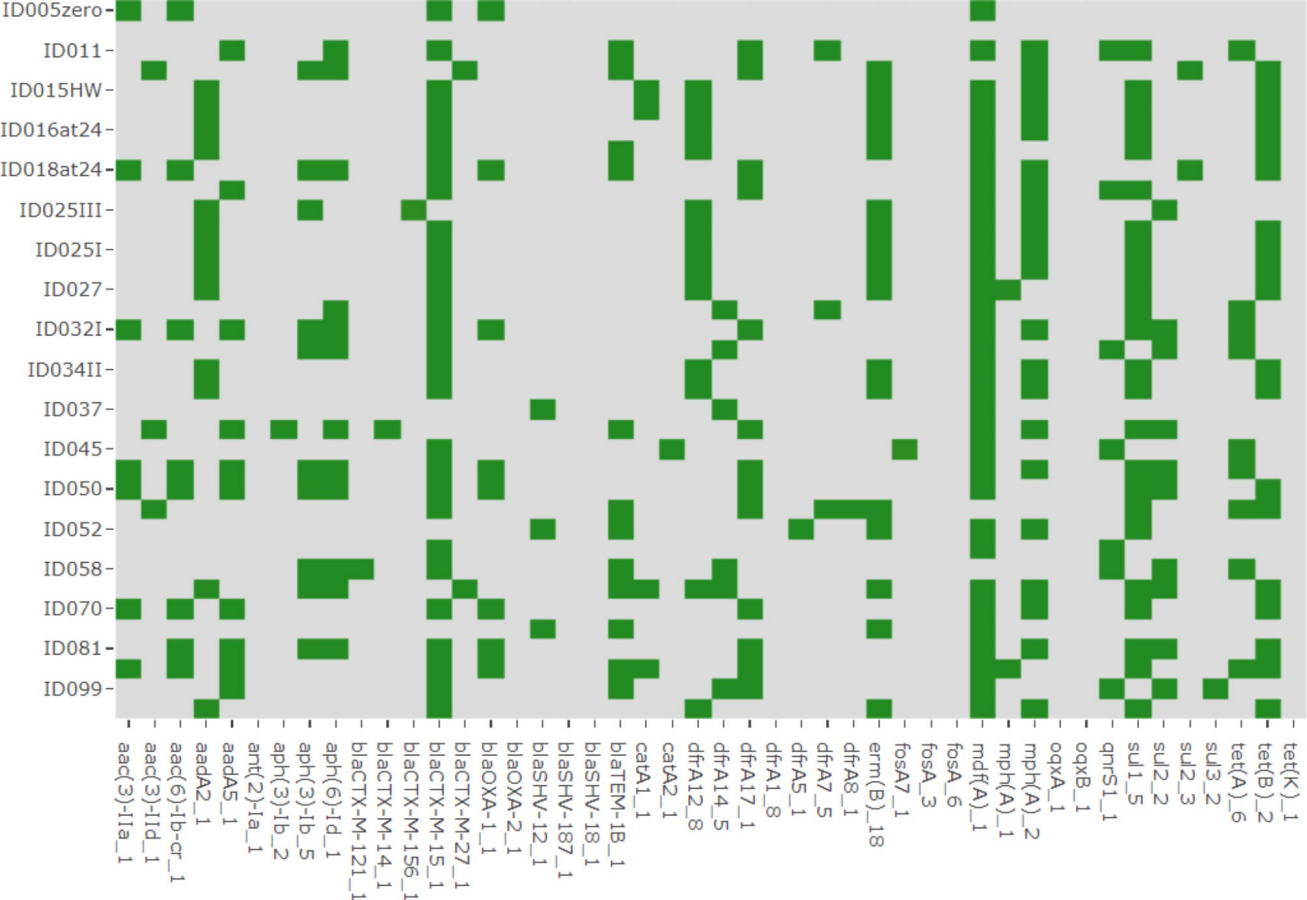
Heat map showing Ugandan *E. coli* predominant genes from ResFinder database

From the Ugandan site, 18/36 (50%) isolates were found to have AMR genes conforming to the set > 90% cut-off for both coverage and identity in the ResFinder database. The most predominant genes identified among the isolates comprised of *bla*_CTX−M−15_ with 11/18(61.1%) which confers resistance to fluoroquinolones, and third-generation cephalosporins, *mdfA* 14/18(77.7%) which confers resistance to chloramphenicol through its efflux pump action, *tet*(B) 9/18 (50%) conferring resistance to tetracyclines and *sul1* 8/18(44.4%) conferring resistance to sulphonamides. The heatmap in Fig. 2 shows the distribution of the respective genes across the isolates. The green color indicates the presence of a gene while the grey shows the absence of that gene per isolate.

**Fig. 3:**
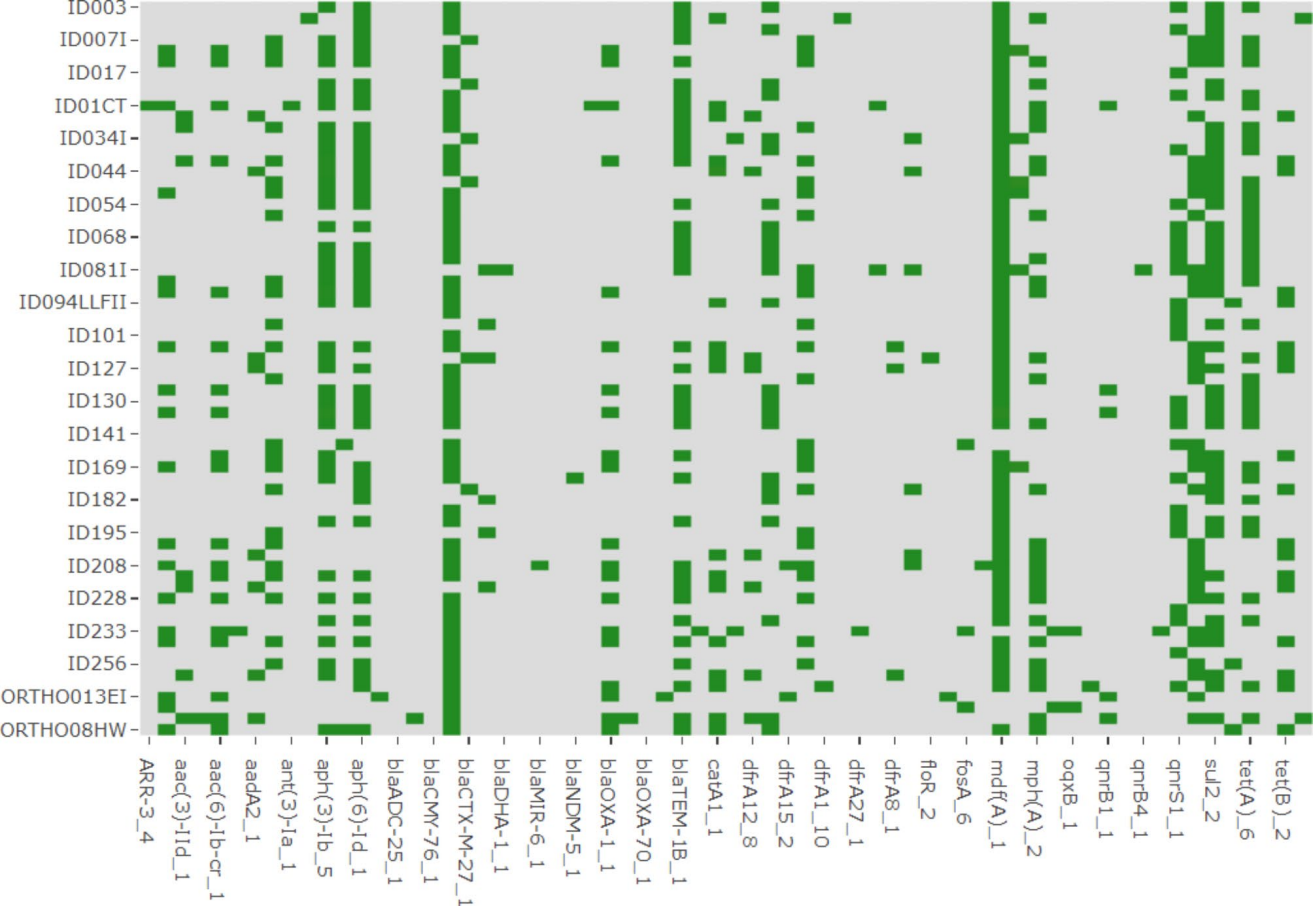
Heat map showing Tanzanian *E. coli* predominant genes from ResFinder database

From the Tanzanian site, 23/67 (34.3%) isolates were found to have AMR genes conforming to the set > 90% cut-off for both coverage and identity in the ResFinder database. The most predominant genes identified among the isolates comprised of *bla*_CTX−M−27_ with 12/23 (52.1%) conferring resistance to fluoroquinolones and third-generation cephalosporins, and *bla*_TEM−1B_ with 13/23 (56.5%) which confers resistance to third-generation cephalosporins, *mdfA*(A) 21/23 (91.3%) which confers resistance to chloramphenicol through its efflux pump action, *tet*(A) 12/23 (52.1%) conferring resistance to tetracyclines and *sul2* 15/23 (65.2%) conferring resistance to sulphonamides. The heatmap in Fig. 3 shows the distribution of the respective genes across the isolates. The green color indicates the presence of a gene while the grey shows the absence of that gene per isolate.

### Multi-locus sequence typing (MLST)

A total of 34/36 (94.4%) isolates for which MLST was performed conformed to the set > 90% cut-off for both coverage and identity for the sequence types (STs). The most predominant *E. coli* from Uganda comprised of the following: ST656 accounting for 9/34 (26.4%) within the isolates, ST206, ST448, ST1193, and ST1284 each accounting for 2/34 (5.8%) within the isolates. The majority of the other STs were; ST131, ST10, ST6438, ST998, and ST38 among others each accounted for 1/34 (2.9%) within the isolates.

**Fig. 4:**
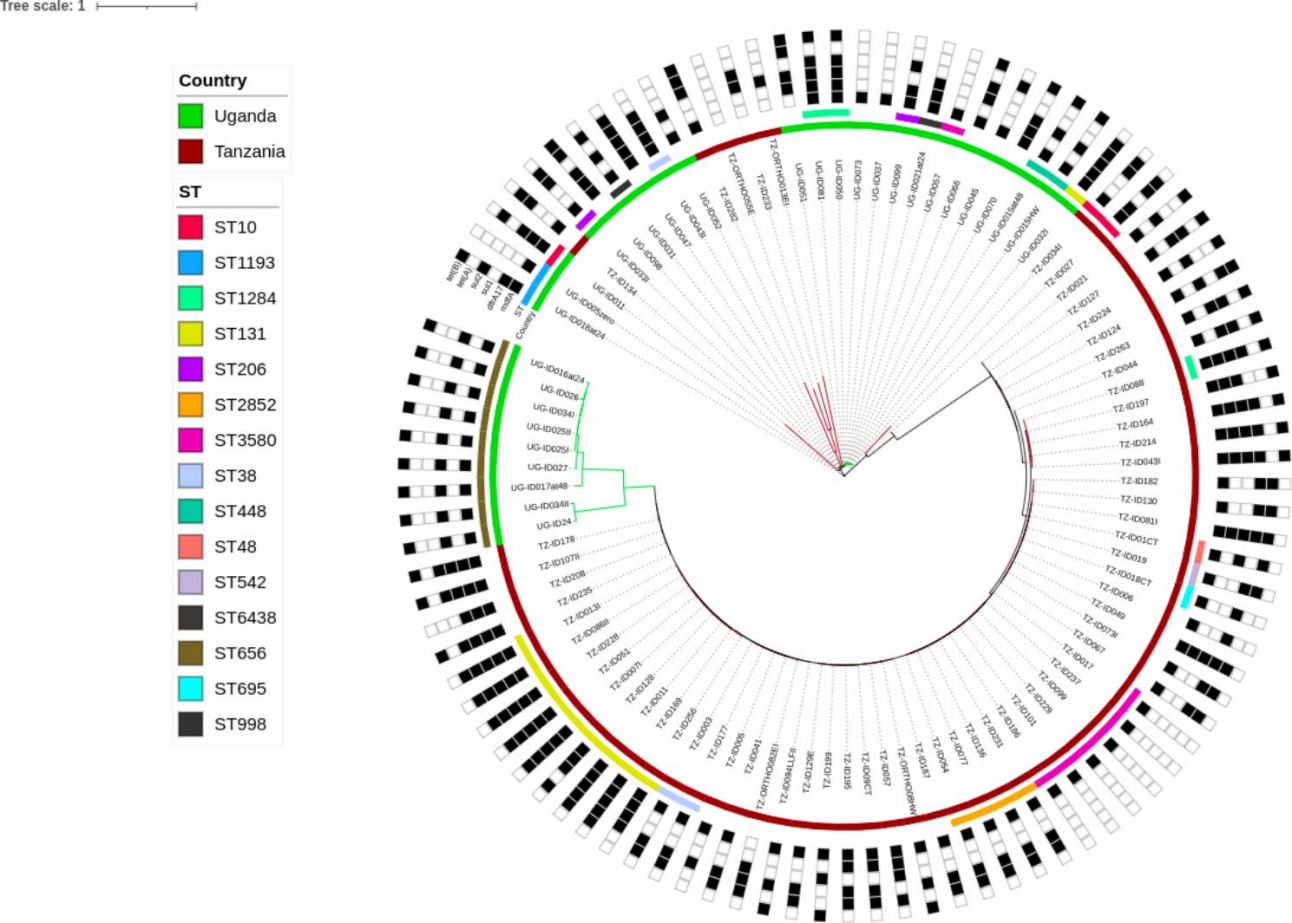
Phylogenetic tree showing the distribution of AMR genes and STs across the two study sites

In comparison with the Tanzanian site, 59/67 (88%) isolates conformed to the set > 90% cut-off for both coverage and identity for the sequence types (STs). The most predominant *E. coli* STs from Tanzania comprised of the following: ST131 accounting for 5/59 (8.4%) within the isolates, ST10, ST2852, and ST167 each accounting for 2/59 (3.3%) within the isolates. Majority of the other STs were: ST3580, ST695, ST542, ST48, and ST38 among others accounted for 1/59 (1.6%) within the isolates. Fig. 4 below shows how the isolates between the two countries cluster in terms of AMR genes and STs.

### Virulence factor detection(VF)

In addition to their AMR genes, the *E. coli* species from the two study sites were found to possess arsenals of virulence factors that are inter-species in nature that is, they originated from other different bacterial species, a clear depiction of horizontal transfer of genetic material between bacteria. The isolates from the two countries had most of their virulence factors identical to those found in *Shigella dysenteriae* Sd197, *Yersinia pestis* CO92, *Salmonella enterica* subsp. enterica serovar Typhimurium str. LT2, *Escherichia coli* O157:H7 str. EDL933, and *Pseudomonas aeruginosa* PAO1. A comprehensive table depicting the distribution of the different virulence factors of the isolates from the two sites is shown in Table 3.

**Table 3:** Distribution of virulence factors among the *E. coli* isolates from Uganda and Tanzania

Virulence Factors (VF)	Physio-biological roles	Isolates	
		Uganda	Tanzania
*gspD, gspE, gspF, gspG*, and *gspF*	General secretion pathway proteins for *Shigella dysenteriae* Sd197	✔	✔
*irp1, ybtU, ybtX* and *iucA*	A yersiniabactin biosynthetic protein for *Yersinia pestis* CO92	✔	✔
*csgF* and *csgG*	Curli production assembly/transport proteins for *Salmonella enterica* subsp. enterica serovar Typhimurium str. LT2	✔	✔
*gspC, gspH*, and *gspI*	General secretion pathway proteins C for *Shigella dysenteriae* Sd197	✔	✔
*espL1, espL4, espR3, espR4, espX1, espX4, espY1*, and *espY3*	Type III secretion system effectors for *Escherichia coli* O157:H7 str. EDL933	✔	✔
*astA*	A heat-stable enterotoxin 1 for *Escherichia coli* O44:H18 042	✔	–
*eltA* and *eltB*	Heat-labile enterotoxins from human for *Escherichia coli*	✔	–
*afaB-I* and *afaC-I*	Chaperone mannose-resistant adhesin proteins for *Escherichia coli* O25b:H4 str. FV9863	✔	
*fepA, fepB, fepC, fepD*, and *fepG*	Ferrienterobactin outer membrane transporter permease Enterobactin for *Escherichia coli* CFT073	–	✔
*ompA*	Outer membrane protein for *Escherichia coli* O18:K1:H7 str. RS218	–	✔
*hlyA, hlyB, hlyC*, and *hlyD*	Hemolysin A for *Escherichia coli* CFT073	–	✔

## Discussion

Control of multi-drug resistant infections is fundamental in reducing the disease burden and costs incurred while treating these pathogens in tandem with the Global Action Plan set by WHO [33] to combat AMR. This study comes in at the right point in time where the scale at which global public health is threatened by the increasing infection rates. In this study, the aim was to explore the genetic determinants that confer AMR from isolates obtained at Mulago National Referral Hospital, Bugando Medical Centre, and their environmental settings. The findings from this study are provocative and inform the dire need to strengthen the existing infection-prevention controls (IPC) together with surveillance and monitoring systems.

This study was predominantly comprised of ESBL organisms; with 36/57 (63.1%) originating from Uganda and 67/85 (78.8%) originating from Tanzania. Previous findings from studies in Uganda reported ESBL *E. coli* prevalence rates of 5.3% conducted between 2006 and 2007 [34], 62% carried out in 2014 [35] at Mulago National Referral Hospital, and between 2015 and 2016 at Kasese Regional Referral Hospital at 62% [36]. Related meta-analysis and systematic review studies from East Africa carried out in hospitals and surrounding communities reported a similar predominance of ESBL-producing *Escherichia coli* and *Klebsiella pneumoniae* [37–39]. The Enterobacteriaceae family has been reported to shape the nosocomial pathogen eco-system because of the plasticity of their genome and their ability to perform inter-species and intra-species incorporation and transfer of drug resistance mediating determinants like plasmids, transposons, insertion sequences, and virulence factors via horizontal gene transfer [40–42].

Detection of SNP-associated mutations in the genes; *RarD*, *yaaA*, and *ybgl* conferring resistances to chloramphenicol, peroxidase, and quinolones from the isolates from both Uganda and Tanzania further depicts how these organisms evolve resistance towards some of the most commonly used antibiotics and antiseptic used for the day-to-day management of clinical cases. A related study highlighted the roles played by these SNPs in the evolution of antimicrobial resistance and in shaping the genome of these organisms [43].

Our results reported very high frequencies for *bla*_CTX−M−15_ accounting for 11/18(61.1%), and *bla*_CTX−M−27_ with 12/23 (52.1%), *bla*_TEM−1B_ with 13/23 (56.5%) of isolates originating from Uganda and Tanzania respectively. These genes are responsible for conferring resistance to penicillin, fluroquinolones, and third-generation cephalosporins (ceftazidime and cefotaxime) which are part of routinely prescribed antibiotics used in the treatment of medical cases within the two sites similar to other previous related studies that reported phenotypic AMR profiles of the same organisms [44]. Tanzania had relatively higher tetracycline resistance gene *tet*(A) with 12/23 (52.1%) as compared to the Ugandan isolates with *tet*(A) with 7/18 (38.8%) prevalence which are in agreement with similar studies performed across six Tanzanian hospitals [45]. We also reported relatively a high prevalence of sulphonamide-resistance conferring genes *sul1* 8/18(44.4%) and *sul2* 15/23 (65.2%) from Uganda and Tanzania respectively. Chloramphenicol resistance gene *mdfA*(A) with 21/23 (91.3%) from Tanzanian isolates and trimethoprim resistance-conferring gene *dfrA17* with 8/23 (34.7%) in Ugandan isolates were also observed within the two cohorts. The authors propose that the high resistance observed in the majority of over-the-counter antibiotics can likely be explained by their affordability in the two countries. In contrast, expensive drugs such as piperacillin-tazobactam, amikacin, and carbapenems are less accessible in most drug shops within the two study sites. Consequently, these expensive drugs are less likely to be misused, leading to lower detected resistance levels. These findings sound a very big alarm about the potential dangers such pathogens can cause to the general public health and call for the need to scale up the microbiology laboratory capacity as a way of guiding antimicrobial agent prescription. Data from well-established laboratory facilities will shape and strengthen AMR surveillance, IPC protocols within community-based settings and healthcare facilities, and regulation of drug prescriptions from drug outlets and pharmacies.

The largest portion of sequence types isolated from Tanzania belonged to the ST131 accounting for 5/59 (8.4%) of the total isolates while Uganda was represented with 1/34 (2.9%) for ST131 of the total isolates. These sequence types associated with extra-intestinal infections have been reported to be rapidly spreading as high-risk clones in Europe and worldwide due to increased AMR [46–49]. Some other sequence types like ST206 have been reported to be associated with colistin-resistance conferring isolates from a study in China [50]. These findings reiterate the dangers these organisms impose on the public health system and call for immediate interventions [51].

The presence of virulence factors like *Shigella dysenteriae* Sd197 (*gspD, gspE, gspF, gspG*, and *gspF*), *Yersinia pestis* CO92 (*irp1, ybtU, ybtX*, and *iucA*), *Salmonella enterica* subsp. enterica serovar Typhimurium str. LT2 (csgF and csgG), *Shigella dysenteriae* Sd197 (gspC, gspD, gspE, gspF, gspG, gspH, and gspI), and *Pseudomonas aeruginosa* PAO1 (*flhA, fliG*, and *fliM*) in isolates from both Tanzania and Uganda reported by this study depict a classic case of inter-species genetic-determinant element transfer. Multiple studies have reported how the horizontal gene transfer, a process through which genetic information can be acquired from the environment to a bacterium or from one bacterium to another through other mechanisms other than chromosomal inheritance consequently shaping pathogen virulence evolution [52–56]. This study provides strong evidence regarding the acquisition of a set of rather queer virulence factors like *Yersinia pestis* CO92 among the *Escherichia coli* isolates originating from a somewhat deadly plague-causing bacteria similar to what has been reported by a study in India [57].

The ingenious advent of bioinformatics platforms like rMAP [10] used to profile all these virulence elements within the isolates in one go provides a comprehensive way of analyzing WGS data because of its easy installation, usage, and applicability, especially in low-income settings where high-performance computing infrastructure is limited. In our opinion, it also bridges and fills the missing link between the rapidly embraced field of WGS and conventional microbiology while providing high-resolution, and shorter result-generating turnaround times for the genomes of MDR pathogens. It is on these grounds that we recommend this tool to be adopted as a continuous monitoring and surveillance software for monitoring the antimicrobial resistance gene trends, plasmids, virulence factors, and MLSTs within community and healthcare settings for Uganda, Tanzania, and Africa as a whole.

## Conclusion

This study shows a notable abundance and diversity of the AMR-conferring elements shaping the genomic structure and survival of *E. coli* isolates from the hospital and clinical settings in Uganda and Tanzania. The findings from this study are provocative and inform the dire need to strengthen the existing infection-prevention controls (IPC) and adoption of WGS alongside conventional microbiology as a way of building genomics surveillance and monitoring capacity in East Africa.

### Limitations

Due to financial limitations, our study relied exclusively on WGS short-read data for deductions. Introducing long-read sequencing such as Nanopore sequencing requires a low quantity of input DNA and is suitable for low-concentration samples and in conjunction with short-read might have enriched our understanding of the AMR-conferring elements elucidated in our research. Hybrid long-short-read sequencing approaches offer a powerful tool in microbial genomic research. By combining the strengths of both long-read and short-read sequencing, hybrid approaches offer improved accuracy, read length, and cost-effectiveness.

## Data Availability

All data generated or analyzed during this study are included in this published article. All source code for the rMAP pipeline, installation instructions, and implementation can be accessed via GitHub (https://github.com/GunzIvan28/rMAP). The source code is available on GitHub under the GPL3 license. Questions, bugs, or any other issues can be filed as GitHub issues. Although rMAP itself is published and distributed under a GPL3 license, some of its dependencies bundled within the rMAP volume are published under different license models. The genomic raw reads files from this study are publicly available at the Sequence Read Archive (SRA) of the National Center for Biotechnology Information (NCBI) under the study BioProject ID: PRJNA840888.

## Abbreviations

AMR: antimicrobial resistance
CUHAS: Catholic University of Health and Allied Sciences
ESBL: extended-spectrum beta-lactamase
HGT: horizontal gene transfer
IPC: infection and prevention control
MDR: multi-drug resistance
PDR: pan-drug resistance
rMAP: Rapid Microbial Analysis Pipeline
STs: sequence types
